# Reducing metabolic burden in the PACEmid evolver system by remastering high‐copy phagemid vectors

**DOI:** 10.1049/enb2.12021

**Published:** 2022-05-20

**Authors:** Beth India Davenport, Jure Tica, Mark Isalan

**Affiliations:** ^1^ Department of Life Sciences Imperial College London South Kensington Campus London UK

## Abstract

Orthogonal or non‐cross‐reacting transcription factors are used in synthetic biology as components of genetic circuits. Brödel et al. (2016) engineered 12 such cIλ transcription factor variants using a directed evolution ‘PACEmid’ system. The variants operate as dual activator/repressors and expand gene circuit construction possibilities. However, the high‐copy phagemid vectors carrying the *cIλ* variants imposed high metabolic burden upon cells. Here, the authors ‘remaster’ the phagemid backbones to relieve their burden substantially, exhibited by a recovery in *Escherichia coli* growth. The remastered phagemids' ability to function within the PACEmid evolver system is maintained, as is the cIλ transcription factors' activity within these vectors. The low‐burden phagemid versions are more suitable for use in PACEmid experiments and synthetic gene circuits; the authors have, therefore, replaced the original high‐burden phagemids on the Addgene repository. The authors’ work emphasises the importance of understanding metabolic burden and incorporating it into design steps in future synthetic biology ventures.

## 1 INTRODUCTION

Well‐characterised transcription factors (TFs) and their DNA operator binding sites are widely used within synthetic gene circuits. They are arranged in such a way as to carry out user‐defined functions, to give predictable gene expression patterns as outputs and are becoming increasingly useful for biomedical and biotechnological applications. The first dynamical synthetic circuits were built using just a few TFs and their operators, such as the genetic toggle switch and repressilator systems [1, 2]. Although increasing numbers of TFs are repurposed for synthetic biology year‐by‐year, the field is still running short of diverse, orthogonal TF sets that are required for increasing circuit complexity [3, 4, 5, 6, 7, 8]. Expanding this toolkit through engineering TFs with different properties and orthogonal activities is, therefore, a topic of current focus [3, 7, 9, 10, 11, 12].

Brödel et al. [3] addressed the need for novel TFs by building a system for directed evolution called phagemid‐assisted continuous evolution (PACEmid). It is inspired by the original PACE system [13, 14] and utilises the M13 bacteriophage life cycle [15, 16] to evolve new and mutually orthogonal TF‐operator pairs [3]. The PACEmid system involves three plasmids, one of which is a phage‐packaged phagemid, containing the gene to be evolved, and allowing combinatorial libraries to be cloned more easily. The phagemid is the focus of this study. As an essential phage gene is under conditional control and is only expressed when an evolved TF binds a new operator sequence, the new TF‐operator pair is selected and enriched in the phage population over time. The evolver system is described in detail by Brödel et al. [3].

**FIGURE 1:**
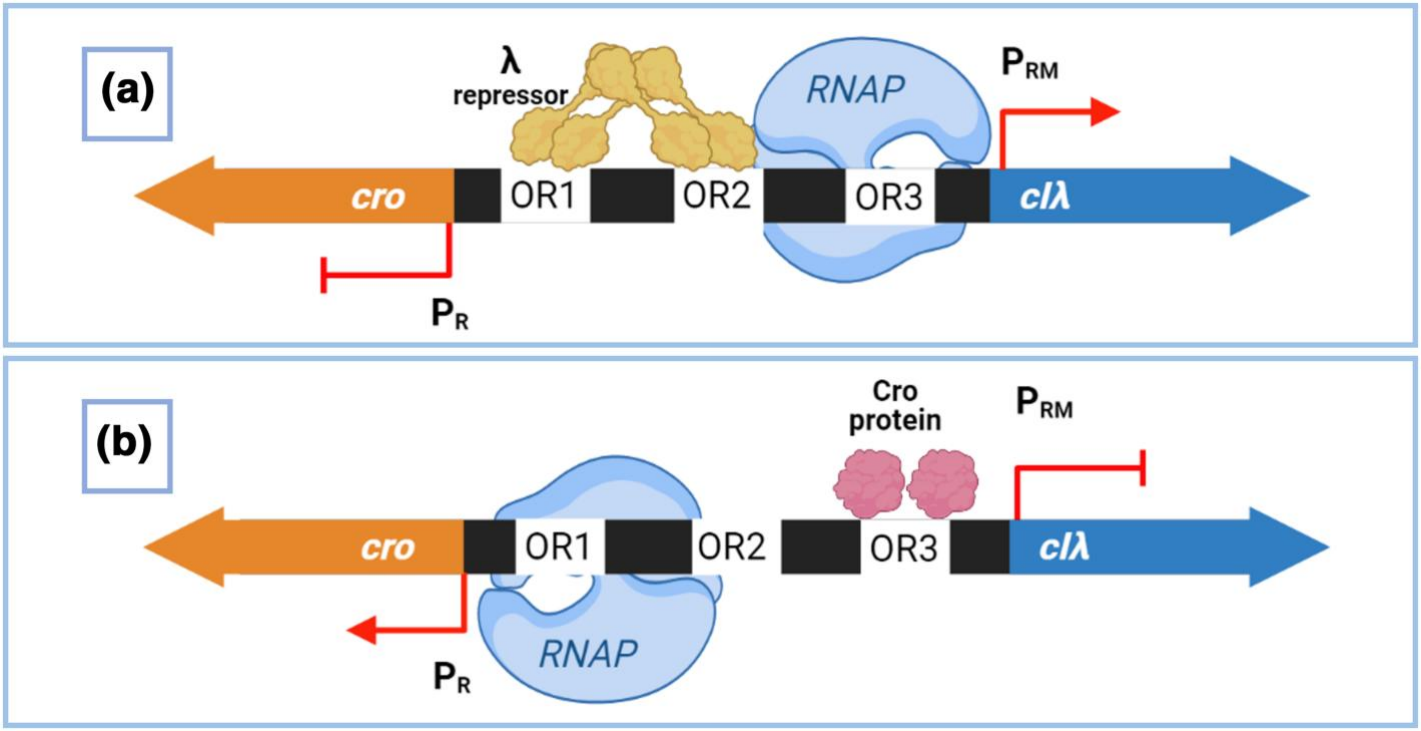
The bacteriophage λ genetic toggle switch. (a) When the repressor cIλ dominates, its dimers recognise and bind to the operator sequences OR1 and OR2 cooperatively. This (i) represses *cro* expression from P_R_ by blocking RNA polymerase access and (ii) recruits RNA polymerase at P_RM_ to activate *cIλ* transcription [17]. (b) When cro dominates, it binds OR3. This (i) prevents RNA polymerase recruitment to P_RM_, repressing *cIλ* expression, and (ii) allows RNA polymerase access to P_R_ for *cro* expression [17]

PACEmid was used to evolve 12 new variants of the bacteriophage λ repressor, encoded by gene *cIλ* [3]. λ repressor harbours dual activator/repressor activity and competes with the transcription factor cro at a natural genetic ‘toggle switch’ in bacteriophage λ′s genome [1, 17, 18, 19]. This switch consists of a bidirectional promoter (P_R_/P_RM_), containing three λ repressor operators (Figure 1). Six new P_R_/P_RM_ promoters with different operator sequences were designed and constructed, and new variants of cIλ were evolved to bind them [3] (Supplementary Figure 1). Brödel et al. obtained 12 novel, completely orthogonal cIλ variants that bind the six new operator sequences: six were evolved from the *cIλ* wildtype gene and six from a *cIoptλ* gene (*cIopt* is a mutant *cI* with stronger RNA polymerase recruitment activity [20]). This enabled two different activating strengths on P_RM_ (Supplementary Figure 2).

The 12 *cIλ* variant‐containing phagemids were deposited on the Addgene public repository, for community use, in 2016 (https://www.addgene.org/browse/article/22969/). However, the phagemids have shown fragility when stored by Addgene and could soon not be grown from their original glycerol stocks. Synthetic gene circuits often impose high metabolic burden on cells [21, 22]. In this case, culture death during storage is likely due to the toxicity of the high‐copy pLitmus (pLit) phagemids [23].

**FIGURE 2:**
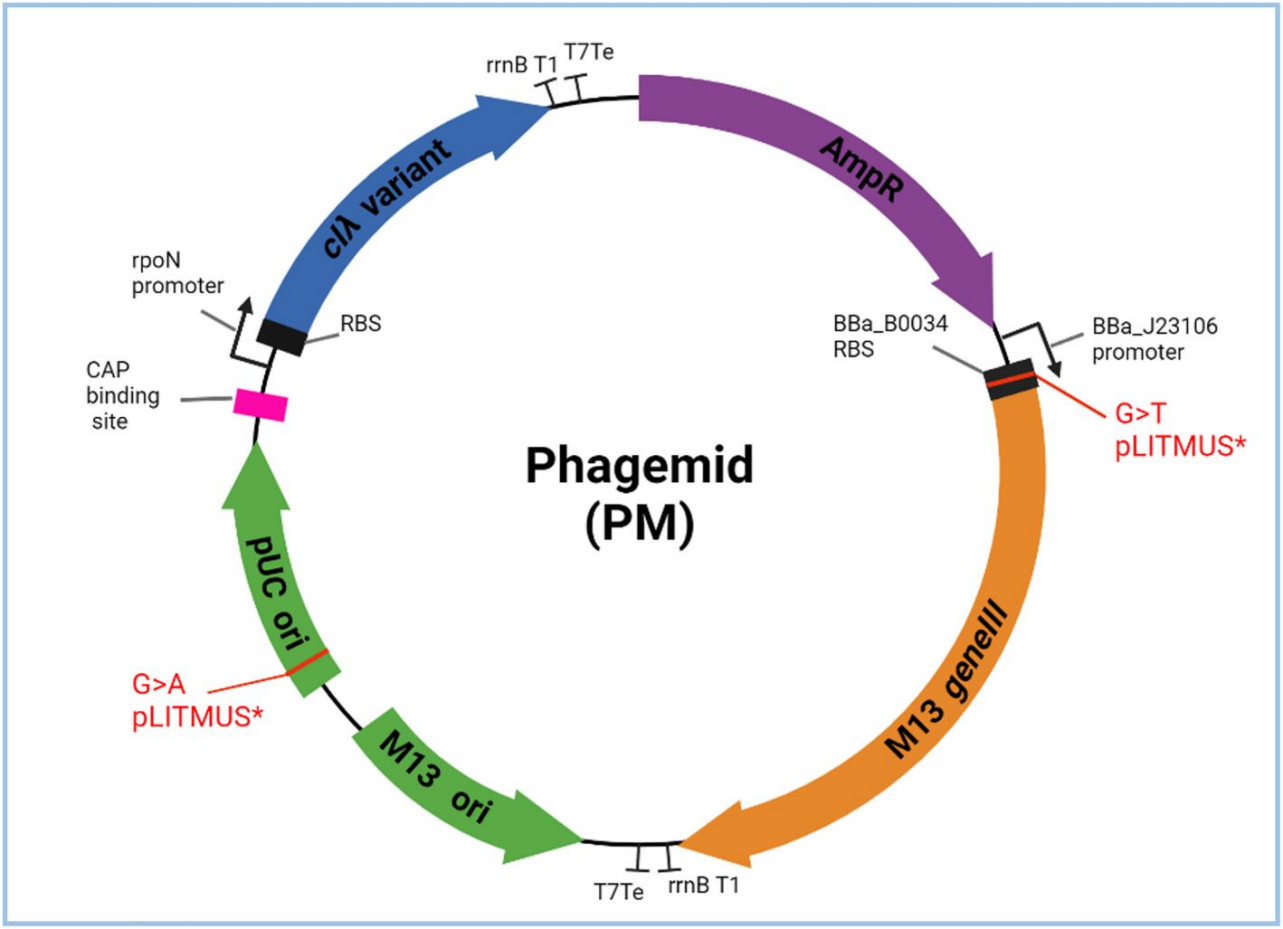
Map of the 12 pLit phagemids. The M13 origin encodes the phage packaging signal. M13 *gIII* is downstream of a strong constitutive BioBrick promoter (J23106) and strong BioBrick RBS (B00034). The *cIλ* variant is downstream of the *rpoN* constitutive promoter and CAP binding site enhancer. *cIλ* and *gIII* have the same BioBrick double terminator, rrnBT1‐T7Te. The ori and *gIII* RBS point mutations introduced by Brödel et al.'s [23] PACEmid experiment, to produce the optimised pLit* vector, are labelled in red

During a recent PACEmid study, an improved pLitmus* (pLit*) phagemid backbone was spontaneously evolved [23]. Preliminary studies suggested that cells containing pLit* show improved growth in liquid culture compared to those bearing pLit [23]. pLit* primarily contains two point mutations compared to pLit: (i) G→T in the M13 *gIII* ribosomal binding site (RBS), and (ii) G→A in the pUC origin of replication (ori) (see Figure 2). Either or both mutations could be driving the apparent reduced metabolic burden. The RBS mutation may work at a translational level to reduce toxic *gIII* expression and/or increase ribosome availability for other cellular proteins [3, 21, 24, 25]. Alternatively, the ori mutation may work to reduce phagemid copy number; many studies have shown ori point mutations driving significant copy number changes [26, 27, 28]. The reduced plasmid copy number has been linked to the improved cell growth [29, 30], possibly through a reduced heterologous gene translation rate through decreasing mRNA abundance [21].

It is important to improve the metabolic burden effects of pLit phagemids for their use in future PACEmid experiments, and for the repurposing of the evolved *cIλ* variants in synthetic gene circuits. Here, we have remastered the 12 pLit cIλ phagemids [3] to pLit* versions and have re‐deposited them on Addgene (see Supplementary Table 1). The remastered phagemids impart improved growth on cells in 2 × YT liquid culture. In addition, reporter assays show that the cIλ variants encoded within the pLit* vectors are expressed and retain binding activity for their corresponding operators. However, the stringent orthogonality between the variants originally evolved by Brödel et al. [3] is lost in the new pLit* context. Lastly, pLit* phagemids can successfully produce phage particles. All the collected data shows that pLit* is compatible with the PACEmid system. Therefore, pLit* phagemids should be prioritised over the burdensome pLit versions in the future.

## 2 RESULTS

### 2.1 Phagemid remastering

Remastering the 12 phagemids from a pLit backbone to a pLit* was carried out through PCR amplification and Gibson assembly. The 12 *cIλ* variants were amplified from the pLit phagemids and assembled with the pLit* backbone of a *cro*‐containing phagemid evolved by Brödel et al. [23]. The Gibson products were transformed into TOP10 *E. coli* cells, and colonies containing each phagemid were cultured and purified. Successful phagemid assembly was verified by restriction digest and gel electrophoresis (as *cro* is ∼0.6 kb smaller than *cIλ*), and the two point mutations and *cIλ* variant sequences were confirmed by DNA sequencing. These 12 pLit* phagemids and their parental pLit phagemids were taken forward for growth, reporter, and phage production assays.

### 2.2 Growth assays

**FIGURE 3:**
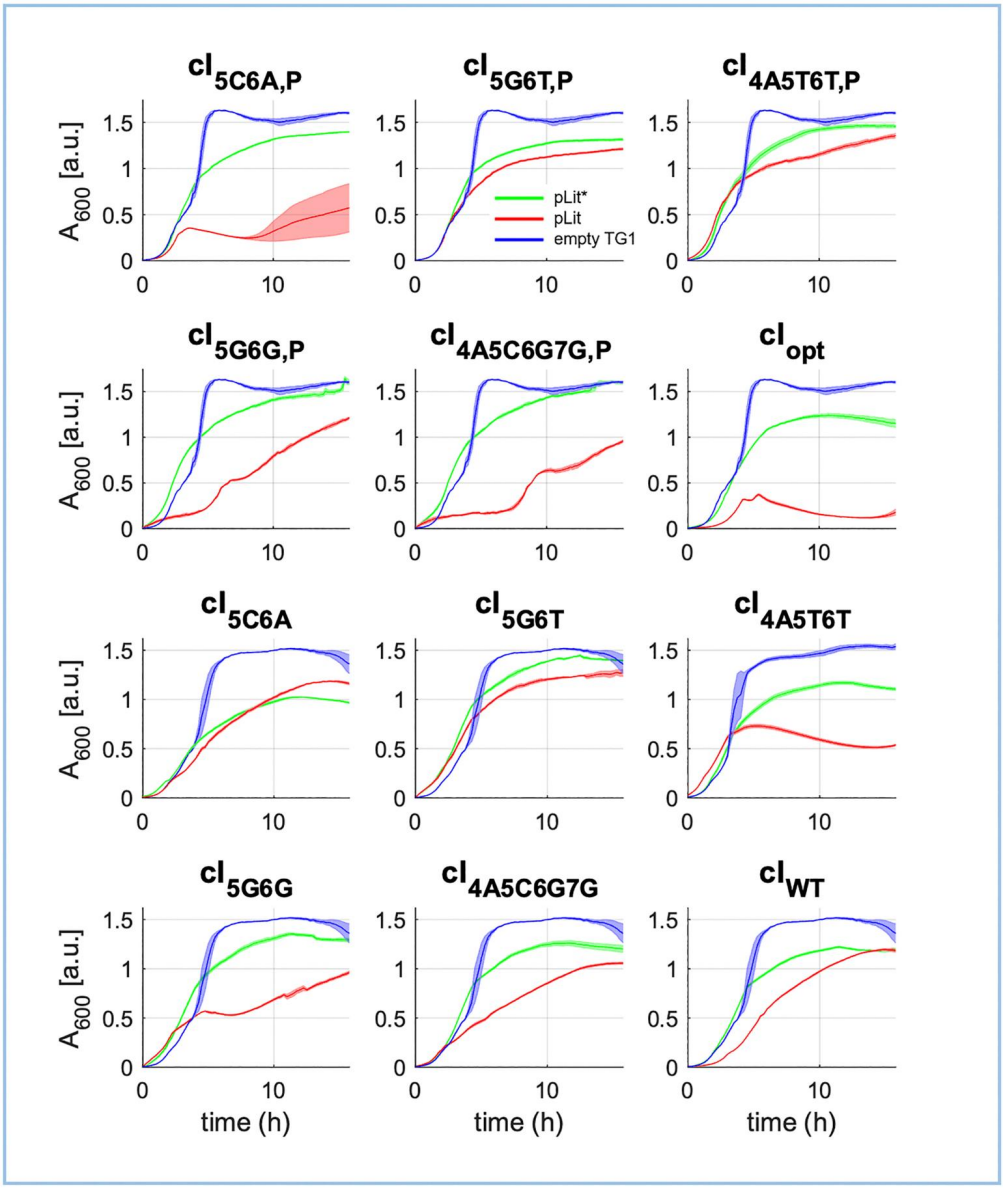
Growth curves of TG1 *E. coli* carrying each of the 12 pLit or pLit* phagemids. Each graph compares the absorbance at 600 nm of cell cultures carrying a pLit* phagemid (green) with cells carrying the parental pLit version (red) over 16 h in 2 × YT culture. Untransformed cells were used as a control (blue). Shaded error bars show the standard error of the mean of three technical replicates

TG1 *E. coli* cells were transformed with pLit* or pLit phagemids to investigate the imparted growth effects of each in 2 × YT culture. Growth curves were plotted from absorbance measurements during a 16 h plate reader experiment. Figure 3 shows the growth curves of untransformed TG1 cells, compared to those carrying a pLit* phagemid or the parental pLit version. Eleven of the 12 pLit* phagemids show substantially improved growth (green) compared to their parental pLit (red). Though cells carrying the pLit*‐cI_5C6A_ phagemid do not show improved final culture density compared to pLit‐cI_5C6A_, they do show a higher initial growth rate, which is more similar to untransformed cells' growth (blue). Importantly, all pLit*‐containing cells' growth patterns are consistently uninterrupted and monophasic, with similarly high growth rates that are only slightly lower than untransformed TG1 cell growth. Contrastingly, pLit‐containing cells show a diverse array of initial growth rates and final culture densities. Therefore, growth assays indicate that metabolic load is relieved, at least to some extent, with pLit* phagemids.

pLit‐containing cell populations show reduced initial growth rate and/or reduced final culture density; moreover, most growth curves are interrupted and some are biphasic. The cell populations carrying pLit‐cI_5C6A,P_, pLit‐cI_4A5C6G7G,P_, pLit‐cI_5G6G_ and pLit‐cI_5G6G,P_ are particularly disturbed, and cells containing pLit‐cI_opt_ even ‘crash’ after 5 h of growth. Despite the diversity in growth patterns, each pLit phagemid differs from the others by only a handful of point mutations within the *cIλ* variant that alter DNA‐binding specificity. Potentially, each cIλ variant could be binding different arrays of off‐targets within the *E. coli* genome; off‐target sites effecting expression of genes playing a role in metabolism could drive the observed growth variations [24]. However, the growth assays alone are insufficient to conclude whether cIλ variants (and wild‐type) are displaying off‐site activity. Notably, growth diversity is also observed within the three technical replicates of pLit‐cI_5C6A,P_‐containing cells. In addition, *cIλ* variants contained in pLit* phagemids do not exhibit such variation during growth assays. Hence, pLit‐containing cell growth diversity could also be due to the stochastic environment of cells experiencing high metabolic burden [22, 31].

*E. coli* can lose a plasmid through segregational instability or plasmid de‐amplification in a non‐selective environment [32, 33, 34, 35]. During these growth assays, ampicillin within the bacterial cultures should prevent cells discarding the metabolically burdensome pLit phagemids. However, the *AmpR*‐encoded *ß*‐lactamase enzyme, present on pLit, slowly metabolises the antibiotic. It, therefore, may be that the second phase of the diphasic growth of pLit‐cI_5C6A,P_, pLit‐cI_4A5C6G7G,P_, pLit‐cI_5G6G_ and pLit‐cI_5G6G,P_‐containing cells, where their growth seems to be recovering, is due to culture ampicillin concentration reaching a non‐toxic threshold, enabling high‐burden phagemid loss from the cell population [32, 33, 34, 35, 36].

All phagemid‐containing cells have lower final culture densities than untransformed cells. This is likely due to heterologous gene expression sequestering some transcriptional and translational cellular resources for growth [21, 24, 25, 31, 34]. Reduction in cellular expression resources is also hypothesised to be driving the reduced growth of pLit‐containing cells compared to pLit*‐containing cells. Improved growth of pLit* cells is due to either or both of (i) decreased RBS strength and consequently *gIII* translation rate, due to the RBS mutation and (ii) decreased phagemid copy number, driven by the ori mutation, decreasing cellular phagemid mRNA content. Such mutations that relieve the stress of heterologous gene expression [16] in transformed cells are expected from PACEmid evolution experiments, which blindly select for variants with better growth and phage‐production dynamics.

### 2.3 RBS strength and phagemid copy number analysis

Many synthetic biology studies have shown that a cell's translational resources, specifically the free ribosome pool, are the critical factor for optimal cell growth [21, 37, 38, 39]. Increasing RBS strength of heterologous mRNA transcripts has been modelled to reduce the free ribosome pool in a cell, thus inhibiting cellular growth [21]. Hence, RBS strength plays an important role in determining the success of synthetic gene circuits within cells, where an uninhibited metabolism is important for a healthy cell and for a well‐behaving circuit [24, 40, 41, 42, 43]. pLit *gIII* is constitutively expressed from a high‐copy plasmid under a medium strength promoter (BBa_J23106) and translated under a strong RBS (BBa_B0034), the combination of which is likely to result in very high expression levels and burden (Figure 2) [2, 3]. In fact, when a *gIII*‐only variant of pLit is constructed, where the *cI* gene is deleted, it cannot be cloned into *E. coli* because its toxicity prevents the growth of cells. Thus, the strong expression of *gIII* from pLit is sufficient to cause cell death and is likely to be one of the main causes for toxicity. This is not true for pLit*, where a *gIII*‐only variant can be cloned easily and shows good growth in liquid media and on agar. This also reveals a competition for resources between *cI* and *gIII*. The addition of the *cI* cassette sequesters some resources away from *gIII*, enabling the cells to survive. Thus, the original design of the pLit constructs is very close to the toxic regime, where the cells cease to grow, and needs to be redesigned. The following section will look at the roles of the two pLit* mutations that were spontaneously evolved for reduced burden [3].

To predict the effect of the pLit* RBS mutation on *gIII* translation rate compared to pLit, Salis et al.'s [44] highly‐accurate RBS calculator was used [41, 45]. This employs a Gibbs free energy model to quantify the thermodynamic interaction between an mRNA molecule and a ribosome and outputs translation initiation rates for all start codons in the mRNA input sequence on a proportional scale, ranging from 0.001 to 100,000 arbitrary units (au).

**FIGURE 4:**
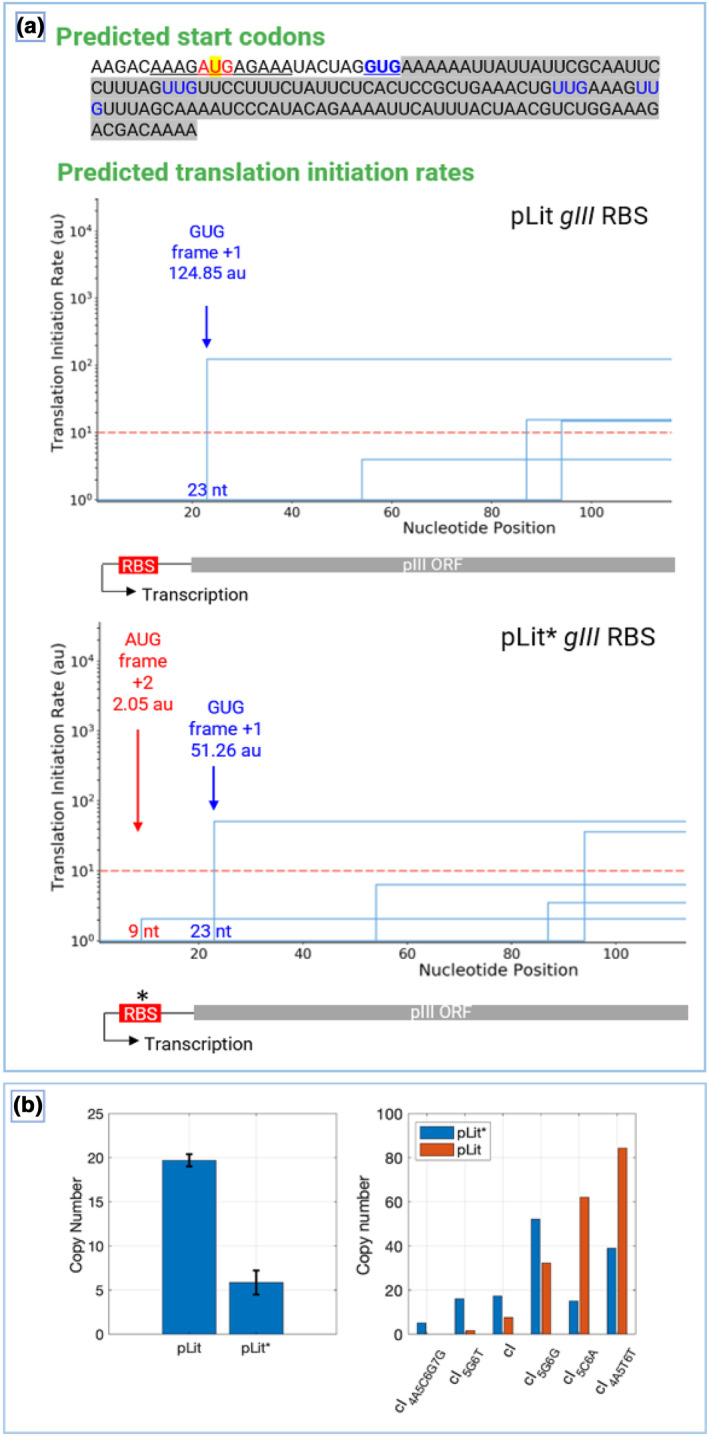
pLit* RBS and ori mutation analysis. (a) pLit versus pLit* RBS analysis, calculated by Salis et al.'s [44] RBS calculator. mRNA sequence input of pLit* (top section, pLit input is identical except for a G in place of the highlighted U) depicts the *gIII* open reading frame (ORF) (grey highlighted), the *gIII* RBS (underlined, with the pLit* point mutation yellow highlighted), and all predicted start codons around the *gIII* start site (blue, with the actual GUG start codon of *gIII* bold underlined). Predicted translation rates of each start codon are plotted below this (bottom sections), with the rate and frame of the GUG start codon indicated (blue arrows), and rate and frame of a new start codon AUG introduced by the pLit* point mutation (*) indicated (red arrow). (b) Left panel depicts copy number analysis of empty pLit and pLit* phagemids in mid‐exponential phase. Error bars show standard deviation (*n* = 3). Right panel depicts copy numbers of pLit and pLit* phagemids bearing each of the six cI variants in mid‐exponential phase

Figure 4a illustrates that the pLit* mutation has a substantial predicted effect, reducing the *gIII* translation rate by ∼60%. Notably, the pLit* G→T point mutation inactivates the strong *gIII* RBS. It also introduces a new, out‐of‐frame AUG start codon, which may contribute to the decrease in pLit* *gIII* translation rate by competing with the weaker in‐frame GUG start codon [46]. The original RBS sequence of pLit *gIII* mRNA predicted by Brodel et al. [23] is AAAGAGGAGAAA. This has a high A/G content (12/12) that is 7 nucleotides upstream from the correct start codon GUG for *gIII* and is downstream of the promoter Pribnow box, all criteria of a strong RBS [47, 48]. The new, pLit* out‐of‐frame AUG start codon requires a separate RBS for translation, predicted by us to be the sequence AGCGAAGACAAA. This is also high in A/G content (10/12), downstream of the promoter Pribnow box, and 2 nucleotides upstream of the new start codon AUG. It also shares high similarity of sequence with other known RBSs used in synthetic biology, such as the M13Ko7 gene IX RBS and 2.8 RBS from T7 (parts.iGEM.org, [49]). However, the AUG's predicted translation initiation is very low (∼96% less than from the correct GUG *gIII* start codon), so competition with the introduced start codon is less likely to be driving the decreased translation rate (Figure 4a).

This analysis suggests the pLit* RBS mutation as being one determiner for driving the improved *E. coli* growth observed in pLit*‐containing cells compared to pLit‐containing cells by weakening the expression levels of toxic *gIII* (Figure 3). However, the pLit pUC ori is a high‐copy number [26, 50], a factor which has been repeatedly observed to reduce *E. coli* growth rates in synthetic circuits [29, 30, 32, 34, 50, 51]. Therefore, the pLit* ori mutation may also contribute to the improved *E. coli* growth phenotype. The mutation occurs in the first nucleotide of the ori's RNA II pre‐primer sequence [52]. When transcribed, RNA II acts to initiate plasmid replication by binding to a DNA stretch in the pUC ori to form an R‐loop [52]. Hence, the mutation could reduce complementarity or ability of the DNA–RNA complex to form, to slow plasmid replication and consequently reduce phagemid copy number per cell.

To investigate this, pLit and pLit* plasmid copy number analysis was conducted using qPCR, as described by Lee et al. [53]. First, the analysis was performed on empty pLit and pLit* plasmids because *gIII* and *cI* can influence plasmid copy number due to associated toxicities. The cells were grown in 2 × YT liquid culture to mid‐exponential phase, and the plasmid and genomic DNA were co‐purified. The plasmid copy number is the amount of plasmid DNA relative to the amount of genomic DNA. The plasmid DNA was quantified with a plasmid‐specific primer pair that binds to the *ß*‐lactamase antibiotic resistance gene, whereas genomic DNA was measured with a primer pair that binds to the birA genomic locus. A decrease in the copy number was found with pLit* (5.9 mean ± 1.4 SD) compared to pLit (19.7 ± 0.7), where the pLit* copy number was approximately a quarter of that of pLit (Figure 4b, left panel). This difference is statistically significant (*n* = 3, standard deviation error bars, independent sample *t*‐test, and *p* << 0.001).

Second, copy numbers of the pLit and pLit* phagemids bearing each of the six *cI* variants and *gIII* were quantified (Figure 4b, right panel). The data is arranged in ascending order with respect to the pLit copy number to aid its interpretation. There is no clear reduction in plasmid copy number in the pLit* backbone compared to pLit when carrying *gIII* and *cI*: on average pLit* (24.1 ± 17.7) has reduced copy number compared to pLit (31.3 ± 35.2) across the six samples. However, this is not statistically significant, and the pLit* copy number remains relatively high for pLit*‐cI_5G6G_ and is higher than pLit in three cases (pLit*‐cI_5G6T_, pLit*‐cI_WT_, and pLit*‐cI_5G6G_).

The copy number is a result of a complex interplay between many different factors, including burden and toxicity, expression levels, growth rate, and competition for cellular resources [21, 30, 54, 55]. The fact that there is no clear relationship between pLit* and pLit copy numbers in the presence of *cI* and *gIII* is likely due to the intricate interaction between metabolic burden and copy number, where the copy number is likely to decrease in response to burden to relieve its effects on the cell. The pLit copy number, which should be constant, shows high variation between the variants, possibly due to high *gIII* toxicity. The same is true for the pLit* copy number. The highly toxic pLit components are associated with very low copy number, such as pLit‐cI_5G6T_ most clearly but also pLit‐cI_WT_ and pLit‐cI_5G6G_. In fact, pLit‐cI_4A5C6G7G_ was not quantified because it did not grow in liquid culture. For these pLit variants with very low copy numbers, pLit* actually increases their copy number, possibly due to relieved toxicity. In contrast, for pLit‐cI_5C6A_, and pLit‐cI_4A5T6T_ whose copy numbers are high, pLit* reduces their copy numbers, consistent to what is seen with the empty plasmids.

Because of intricate feedback between copy number, expression levels, burden and toxicity, there is no clear trend with the phagemids containing *cI* and *gIII*. However, an overall improvement in their health and growth rate makes the pLit* backbone more suitable than pLit. The pLit plasmids were found to be highly toxic during these experiments: variant pLit‐cI_4A5C6G7G_ did not grow in liquid culture at 37°C. The colonies that did grow after overnight incubation at 37°C were characterised by a small size and an unhealthy morphology. Variants pLit‐cI_WT_, pLit‐cI_5C6A_, and pLit‐cI_5G6G_ showed prolonged growth in liquid culture at 37°C, where they took an additional two to 4 hours to reach mid‐exponential phase compared to the pLit* variants. Such delays and inability to grow were not witnessed with any of the pLit* variants. The morphology of the colonies were healthier, cells grew more densely, and their shape was more rounded. Hence, much of the burden associated to the pLit variants seems to be relieved with pLit*. However, a further reduction in the copy number may be required to reduce the effects that the *cI* variants have on it. Ideally, a copy number that is consistent across all *cI* variants would indicate little burden‐related retroactivity.

### 2.4 Reporter assays

**FIGURE 5:**
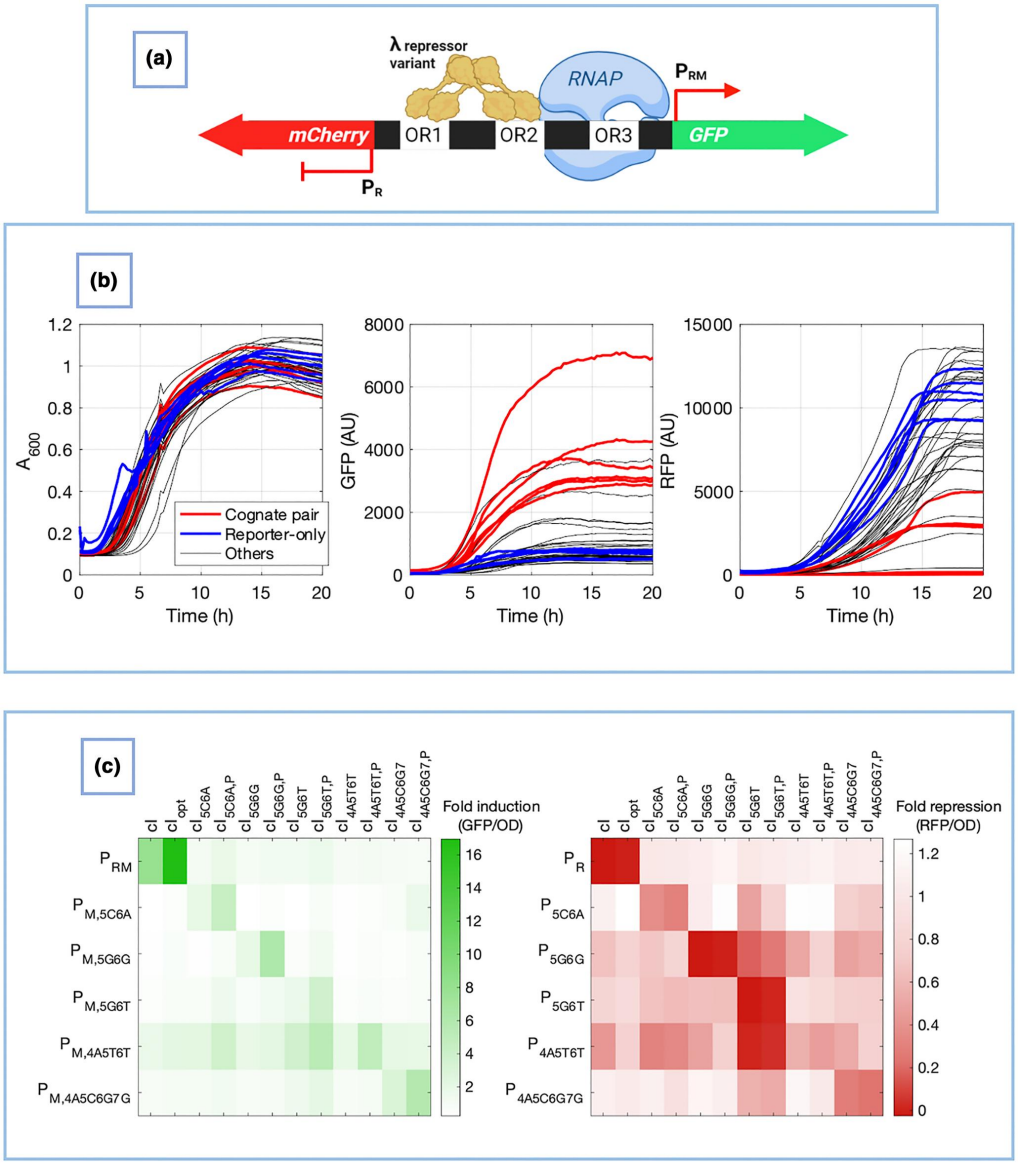
mCherry and GFP fluorescence of TG1 *E. coli* containing different combinations of pLit* phagemids (PMs) and reporter plasmids (RPs). (a) The P_R_/P_RM_ bidirectional promoter structure within the 6 RPs. The operator sequences OR1 and OR2 are different in each RP, recognised and bound by one wildtype cIλ variant and one optimised cIλ variant with high specificity. Without the correct cIλ variant, (i) mCherry is expressed because RNA polymerase has access to P_R_, and (ii) GFP is not expressed because RNA polymerase is not recruited to P_RM_. On cIλ binding, (i) mCherry is repressed from P_R_ as RNA polymerase access is blocked, and (ii) RNA polymerase is recruited at P_RM_ to activate GFP expression. (b) Timeseries plot of the absorbance, GFP fluorescence and RFP fluorescence readings of the six optimised pLit* *cI* variants during the reporter assay. Cognate pairs are plotted in red, six reporter‐only controls are in blue and other non‐cognate pairs are in grey. (c) GFP and RFP fluorescence heatmap of the twelve pLit* phagemid variants at the 20 h timepoint. GFP and RFP were measured for all combinations of pLit* phagemids and RPs in the stationary phase of growth in 2 × YT liquid culture, after the peak response was reached. The data shows the mean of four biological repeats. The data was processed as is described in section 6.6, where values greater than 1 indicate fold‐induction and values between 0 and 1 stand for fold‐repression relative to the reporter‐only measurements

Brödel et al. [3] constructed 6 pJPC12 Reporter Plasmids (RPs), which contain Green Fluorescent Protein (GFP) and mCherry reporter genes on either side of a P_R_/P_RM_ synthetic bidirectional promoter (Figure 5a). To test that the activation of P_RM_ by cIλ variants, and their orthogonality with respect to other P_RM_ sequences, these RPs were used to measure promoter activation for all combinations of pLit* phagemids and pJPC12 RPs. When TG1 cells are transformed with a pLit* and its corresponding RP, cIλ should bind its operator on the RP to repress mCherry at P_R_ and drive GFP expression at P_RM_ (Figure 5a). In contrast, if cells are transformed with a pLit* and a mismatched RP (a ‘non‐cognate’ pair), operator sequences in the RP P_R_/P_RM_ should not be recognised by the pLit* cIλ variant; mCherry should not be repressed, and GFP expression should not be activated.

The GFP and mCherry fluorescence were measured for all combinations of the twelve pLit* phagemids and pJPC12 RPs in 2 × YT liquid culture in the stationary phase of growth. Figure 5b shows reporter assay timeseries data for the six optimised variants. At the 20 h timepoint, the cells are well into the stationary phase of growth and the fluorescence responses are fully induced. Figure 5c shows reporter assay data at 20 h for all variants in heatmap plots. For ten pLit* cIλ variants, correct operator binding and induction are maintained (Figure 5c left panel). Cognate phagemid/RP pairs show substantially increased GFP expression and substantially decreased mCherry expression compared to cells containing non‐cognate pairs in all cases except the pLit*‐cI_4A5T6T_ and pLit*‐cI_4A5T6T,P_ variants. This trend can also be seen in timeseries plots, comparing them with the RP‐only controls (Figure 5b). These results are consistent between biological repeats (Supplementary Figure 5). However, the stringent orthogonality originally observed within the pLit context [3] is absent with the pLit* variants. Heatmaps were also generated for fluorescence readings in mid‐ (A600 = 0.4) and late‐exponential (A600 = 0.8) phases of growth. While the fold‐induction values were slightly different in each case, the trends were the same and nothing new could be concluded from these analyses (data not shown).

Brödel et al. [3] derived a similar heatmap as is shown in Figure 5c with the pLit phagemids, to show stringent orthogonality and a complete absence of cross‐talk between any of the variants. Compared to the study by Brödel et al. [3], there is significant crosstalk between the *cI* variants and loss of orthogonality with the pLit* backbone (Figure 5c). This illustrates the well‐known biological problem of context; selection of constructs under a certain condition does not always port across to another, and therefore parts often require to be re‐optimised on change of context. Therefore, the context of the evolution needs to be chosen carefully [56], and future directed evolution studies should focus on expressing the evolved target genes on lower‐copy plasmids. This will avoid metabolic burden during the evolution process, which can potentially result in the evolution of unsuitable properties, which fail to be maintained in a different cellular context or in the absence of burden. Additionally, engineered circuits are mostly implemented on low‐copy plasmids, so the transcription factor evolution in a high‐copy context may not be suitable [1, 2, 57, 58].

### 2.5 Phage production assays

**FIGURE 6:**
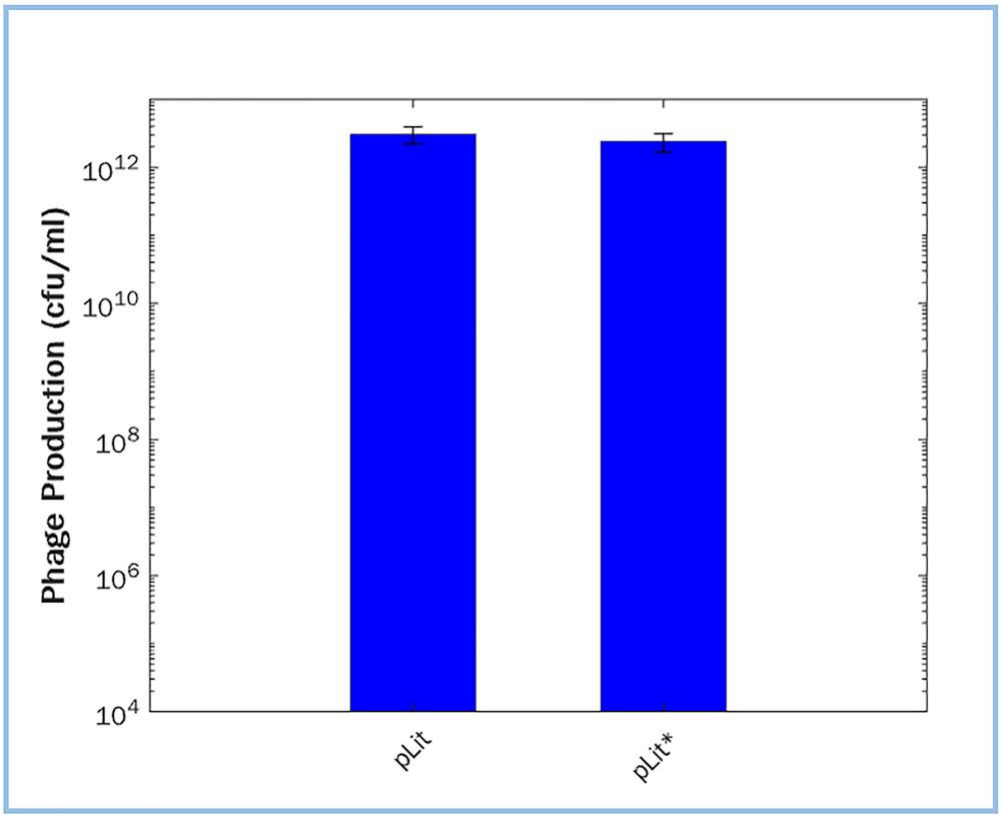
Mean phage titres produced by pLit and pLit* phagemids, calculated from phage production assays in colony forming units per ml (cfu/ml). All 12 pLit* phagemids were tested, along with four pLit phagemids (Supplementary Table 4). The difference between pLit* and pLit titres is non‐significant (independent *t*‐test, *p* = 0.64). Error bars show standard error of the mean

To verify that the M13 phage can be successfully produced with pLit* phagemids in future PACEmid experiments, phage production assays were performed. This involved transforming TG1 cells with a helper plasmid (HP), which contains all phage genes except *gIII*, and with a pLit* phagemid, which contains *gIII* and the phage packaging signal. pLit*/HP‐containing cells were cultured overnight at 30°C for phage synthesis [59], and the phage‐containing supernatant was isolated through centrifugation and sterile filtering. This was also repeated with four pLit parental phagemids. Cells transformed with just a pLit* or HP were used as controls. The phage content of each sample was calculated with a phage titre assay [59]; all pLit* phagemids successfully made phage at high titres, in the order of 10^8^–10^12^ colony forming units per ml, which is comparable with pLit titres from this study (Figure 6) and that of Brödel et al. [3]. In contrast, the negative controls produced no phage, as expected (Supplementary Table 4).

The pLit high copy ori was originally selected for PACEmid to drive high phage production rates, which are advantageous for the system's function [3]. Though the high copy number was expected to increase phage titres, its associated burden has resulted in problems in downstream studies, and may have even negatively impacted the system's performance. Interestingly, observations collected with a simple positive feedback circuit [60] show how a lower‐copy pBR322 ori can produce even higher phage titres compared to the high‐copy pLit ori, due to a reduction in burden (Tica 2018, unpublished data). Although this warrants further investigation, it is likely that the phage‐related mechanisms for viral genome replication do not compete with the bacterial mechanisms for plasmid replication and copy number maintenance [16, 61]. This redundancy makes it unlikely for the phage packaging process to cause reductions in plasmid concentration and consequently negatively impact the rate of phage production. Hence, future phage‐based evolver systems should not assume a high‐copy phagemid as necessary for high phage titres.

## 3 DISCUSSION

This study remastered 12 phagemids, from high to low burden, through introduction of two point mutations; *E. coli* cells containing remastered phagemids show consistent and substantial increases in growth rates and final cell densities in liquid culture, compared to parental phagemids. Our work also confirmed the suitability of the new phagemids for use in PACEmid systems, as phagemids produce phage at high titres when paired with a helper plasmid. Moreover, reporter assays demonstrated that cIλ variants maintain their operator binding and P_RM_ induction activity when within the new phagemid backbones. Hence, the remastered pLit* phagemids versions are recommended to replace the previous pLit variants in future directed evolution experiments. The new phagemids prevent cell growth issues or unwanted mutations [22, 62] in circuit components. Furthermore, they can be more easily subcloned and avoid burden‐associated lack of growth. The 12 remastered phagemids have been deposited to the Addgene repository for such a purpose (see Supplementary Table 1 for Addgene IDs).

The mechanism of burden relief achieved in this study is likely driven by a reduction in heterologous phagemid gene expression. This highlights the importance of avoiding highly burdensome combinations of high‐copy number plasmids, strong promoters and strong RBSs [21, 24, 34, 63]. The study also illustrates how the cIλ variants originally evolved by Brödel et al [3] can lose their stringent orthogonality when ported to other genetic contexts. To engineer more robust components, directed evolution experiments should be designed well outside of ‘burden territory’, in this way avoiding the effects of multi‐layered burden‐related retroactivity on induction dynamics. Designing for lower metabolic burden will prevent the evolution of such context‐dependent properties (Figure 5b) and can be undergone in various ways [64], for example, using software tools to predict transcriptional or translational efficiencies of synthetic constructs [21, 24, 44, 45, 65], or coupling circuit expression with genetically encoded sensors of metabolic burden as feedback controllers [25, 64]. Incorporating such steps into construct design will ensure that the complexity of robust synthetic circuits continues to grow.

Though pLit*‐containing cells show substantial growth improvements to pLit‐containing cells (Figure 3), burden does not appear to be completely relieved. This is particularly noticeable in pLit‐cI_5C6A_, pLit‐cI_WT_, pLit‐cI_opt_ plIt‐cI_4A5C6G7G_ and pLit‐cI_4A5T6T_ containing cells, where final culture density restoration to untransformed cell levels is not complete. Moreover, Ceroni et al.'s [21] cellular capacity experiments show that relatively high synthetic construct burden is needed to reduce cell growth; the lower levels of burden that still significantly impact the dynamics of synthetic systems may not even be detectable at the growth level. Lastly, qPCR assays also suggest that burden is not completely relieved with pLit*; the data shows a *cI* variant‐dependent change in the pLit* copy number, which is however not expected in the complete absence of burden. Perhaps the use of a much lower‐copy ori, such as the PACEmid‐compatible pBR322, could potentially further optimise the system. A low‐copy phagemid should still produce abundant phage titres for PACEmid (Tica 2018, unpublished data). A low‐copy ori may also lead to the evolution of variants that are more easily channelled for downstream use in synthetic circuits, which are very often designed on lower‐copy oris [1, 2, 57, 58]. TF variants' activation/repression dynamics may change if transitioned from a high‐ to low‐copy phagemid; ideally, such essential characteristics are maintained while evolving them for direct transfer into synthetic circuits. The use of low‐copy variants would also overcome the issue of preferentially selecting weak TF variants due to high expression levels associated with high‐copy phagemids.

## 4 CONCLUSION

This study reduced metabolic burden in the PACEmid evolver system [3] by remastering the high‐copy phagemid vectors. Lower burden phagemids containing 12 TF variants have been deposited on Addgene (Supplementary Table 1) and are suitable for use in future synthetic circuitry. The study highlights the importance of avoiding saturating host cell transcriptional and translational resources when designing synthetic constructs [20, 23, 33, 52] and of considering the cellular context within which the construct must function in every step of circuitry design.

## 5 METHODOLOGY

### 5.1 Strains and media

Chemically competent TOP10 *E. coli* were used for DNA cloning and Gibson assembly steps. Electrocompetent TG1 *E. coli* were used for growth, reporter, and phage production assays. Genotypes of these *E. coli* strains used are listed in Supplementary Table 2. Luria‐Bertani agar (Sigma‐Aldrich, L2897‐1KG) and M9 Minimal Media (14 g l^−1^ agar, 1M MgSO_4_, 20% (wt/vol) D‐(+)‐glucose, 1M CaCl_2_, 1M thiamine‐HCl) were used for cell plate growth, and 2 × YT broth (Invitrogen, 22712020) was used to culture the cells. S.O.C. medium (Formedium, SCO0202) was used to recover cells after transformation. Ampicillin (100 μg ml^−1^), chloramphenicol (25 μg ml^−1^), and kanamycin (50 μg ml^−1^) (Sigma‐Aldrich) were added to media where appropriate.

### 5.2 Molecular cloning and plasmid sources

Q5 Hot‐Start High‐Fidelity polymerase (NEB, M0494S) was used for DNA fragment amplification. All PCR reactions were undergone by following manufacturer protocols. PCR products were digested with DpnI at 37°C for 1 h (NEB, R0176 L) and purified with a Monarch DNA gel extraction kit (NEB, T1020 L) from a 1% (w/V) agarose gel (Sigma‐Aldrich, 9012‐36‐6) in TAE medium (Invitrogen, 15558‐042) treated with SYBRSafe (Invitrogen, S33102), run at 90 V for 1 h. Purified fragments were assembled with the Gibson assembly HiFi NEBuilder Kit (NEB E5520S) and transformed into TOP10 cells according to the manufacturer's protocol.

Assembled DNA was purified with the QIAprep Spin Miniprep Kit (Qiagen, 27106) and analysed using a spectrophotometer (Thermofisher, Nanodrop Lite). Successfully assembled phagemids were confirmed by restriction digest with High Fidelity EcoRI (NEB, R3101S) and ScaI (NEB, R3122L) in Cutsmart buffer (NEB, B7204S) and gel electrophoresis (see previous paragraph). All assembled phagemids were sequence verified at both mutation sites (Eurofins Genomics).

All plasmids used in this study are listed in Supplementary Table 1 and maps of the three plasmid classes (reporter, helper and phagemid) are depicted in Supplementary Figure 3. All primers used are listed in Supplementary Table 3. Reporter plasmids used for reporter assays were constructed by Brödel et al. [3]. They contain GFP (GenBank no. KM229386) and mCherry (Uniprot no. X5DSL3), either side of synthetic promoters, the sequences of which are listed in Supplementary Figure 1. The helper plasmid used for phage production assays was sourced from Brödel et al. [23].

### 5.3 Electroporation of *E. coli*

Electrocompetent cells were prepared as in the study by Isalan et al. [66]. Plasmid DNA (0.5 μl at 10 ngμl^−1^) was transferred to 25 μl electrocompetent TG1 cells on ice for 1 min. Cells were then electroporated in a 0.1 cm gap cuvette (Bio‐Rad 1652089) with an exponential pulse of 1.8 kV, 200 Ω, and 25 μF (Bio‐Rad, GenePulser Xcell). Cells were resuspended in 500 μl prewarmed S.O.C. medium and incubated with shaking at 37°C for 90 min. Successfully electroporated cells were selected for by plating on LB agar containing appropriate antibiotics.

### 5.4 Phagemid copy number analysis

*E. coli* were electroporated with each of six phagemids (pLit and pLit*) as described in section 6.3. Samples were grown in 6 ml 2 × YT with shaking at 37°C. DNA samples were prepared from 1 ml culture with the QIAamp DNA Mini kit (Qiagen 51304) following the manufacturer's protocol. The DNA concentration was measured with the Quant‐iT PicoGreen reagent (Invitrogen P7589), and the samples were diluted to 1 ng/μL. The LightCycler 480 SYBR Green I Master Mix was used for the qPCR reactions. The reactions were prepared in a 96‐well plate according to the manufacturer's protocol. Total reaction volume was 20 μL, including 6 μL of diluted DNA and 2 μL of each primer (0.5 μM final concentration). Each sample was run with two primer pairs; the first bound to the bir genomic locus and quantified the amount of genomic DNA, whereas the second bound to the *ß*‐lactamase ampicillin resistance gene (bla) and quantified the amount of plasmid in the samples. The ratio between the plasmid and genome DNA amount is the plasmid copy number. Melting curves were generated after the reactions were completed and showed specific amplification in all reactions and for both primer pairs; the expected product sizes were consistent with the melting curve peaks.

A pBR322 plasmid, containing the binding sites for bir and bla primer pairs, was cloned using the NEBuilder HiFi Assembly Cloning Kit (NEB E5520) and purified with the QIAprep Spin MiniPrep kit (Qiagen 27106). This plasmid was used to prepare a five‐point standard curve with each set of primers, with 10‐fold dilutions between 10^9^ and 10^5^ copies. The standard curves and details of the methodology are shown in Supplementary Figure 4. Standard curves were used to convert the Ct values to the copy number of genomic and plasmid DNA, determined with the bir and bla primer pairs, respectively. The copy number of bla was then divided by that of bir to obtain the plasmid copy number. Phagemid pLit‐cI_4A5C6G7G_ was not quantified because it did not grow in liquid culture.

### 5.5 Growth assays

Electrocompetent TG1 *E. coli* were transformed by electroporation and selected overnight on LB agar containing appropriate antibiotics. Single colonies were cultured to OD_600_ 0.4–0.6 in 5 ml of appropriately antibiotic supplemented 2 × YT, at 37°C and 250 rpm shaking. Cultures were diluted to OD_600_ 0.01 with 2 × YT and appropriate antibiotic and added to a 96‐well plate (Greiner Bio‐one, 655090) for growth and reporter assays in a Tecan SPARK plate reader, with three 150 μl technical replicates for each cell sample. To prevent evaporation, 2 × YT media blanks surrounded the sample wells. 600 nm absorbance was measured for each well every 10 min for 16 h, at 37°C, with shaking between readings (2 mm amplitude and 240 rpm).

Data analysis was conducted on MATLAB R2020a (Mathworks). Absorbance measurements were background corrected by subtracting 2 × YT media blank readings. The mean and standard error of the mean for the three technical replicates were calculated and plotted.

### 5.6 Reporter assays

TG1 *E. coli* were electroporated with all combinations of pLit* phagemids and pJPC12 reporter plasmids as described in Section 6.3. Colonies were resuspended in the 96‐well plate containing 150 μL of 2 × YT (Sigma‐Aldrich Y1003) with appropriate antibiotics. Four colonies were assayed in separate wells for each of the phagemid‐reporter combinations on two different days. The plate was transferred to the Tecan f200pro microplate fluorescence reader. Plates were incubated shaking (mode: orbital, amplitude: 2 mm, and frequency: 280 rpm) at 37°C. Measurements of absorbance at 600 nm and fluorescence (GFP ex: 485/20 nm; GFP em: 535/25 nm; GFP gain: 20; RFP ex: 590/20 nm; RFP em: 625/35 nm; RFP gain: 38) were taken every 10 min for 20 h. After 20 h, the cells grew well into stationary phase and the fluorescence responses were fully developed and showed little change in time. The fluorescence at the final timepoint subtracted the blank 2 × YT readings and normalised to the absorbance at 600 nm. The data presented in the heatmaps was further normalised to the reporter‐only absorbance‐corrected fluorescence readings. Values greater than 1 indicate a fold‐increase, whereas values between 0 and 1 indicate a fold‐decrease relative to the reporter‐only readings.

### 5.7 Phage production assays

Electrocompetent TG1 *E. coli* were transformed by electroporation and grown overnight in 2 × YT containing appropriate antibiotic at 30°C and 220 rpm. Cultures were centrifuged at 8000 g for 10 min, and the supernatant was sterile filtered (0.22 μm pore size, Millex‐GV) to produce phage stocks.

Brödel et al.'s [59] phage titre assay was used to quantify phage stock concentrations. Empty TG1 cells were plated on M9 minimal media to ensure F‐pilus expression (necessary for phage infection) and incubated at 37°C for two days. A single colony was cultured to OD_600_ 0.4–0.6 in 2 × TY, at 37°C and 250 rpm shaking. Phage stocks were diluted to 10^−4^, 10^−6^, and 10^−8^ in 2 × YT media, and 100 μl was used to infect 900 μl cultured cells. Transduced cells were plated on LB agar supplemented with ampicillin and incubated at 37°C overnight. Colony counting was undergone to derive colony forming units of phage particles per ml of phage stock.

## CONFLICT OF INTEREST

The authors declare no competing financial interests.

## Supporting information

Supplementary Material

## Data Availability

Data available on request from the authors.
