# Supplementary material for: Reducing metabolic burden in the PACEmid evolver system by remastering high‐copy phagemid vectors
Source: Eng Biol. 2022 May 20;6(2-3):50–61. doi: 10.1049/enb2.12021 (PMC9996709; doi:10.1049/enb2.12021)
Supplement: Supplementary file 1 — Supplementary Material [file ENB2-6-50-s001.docx]

**Supplementary Table 1**: **Plasmids used in this study.**

| **Plasmid** | **Class** | **Report Name** | **Source** | **Addgene ID** |
| --- | --- | --- | --- | --- |
| pLITMUS-rpoN-cI4A5C6G7G,P-J23106-geneIII | Phagemid | pLit-cI_4A5C6G7G,P_ | Brodel et al. [2] | 80864 |
| pLITMUS-rpoN-cI4A5T6T,P-J23106-geneIII | Phagemid | pLit-cI_4A5T6T,P_ | Brodel et al. [2] | 80863 |
| pLITMUS-rpoN-cI5G6T,P-J23106-geneIII | Phagemid | pLit-cI_5G6T,P_ | Brodel et al. [2] | 80862 |
| pLITMUS-rpoN-cI5G6G,P-J23106-geneIII | Phagemid | pLit-cI_5G6G,P_ | Brodel et al. [2] | 80861 |
| pLITMUS-rpoN-cI5C6A,P-J23106-geneIII | Phagemid | pLit-cI_5C6A,P_ | Brodel et al. [2] | 80860 |
| pLITMUS-rpoN-cIopt-J23106-geneIII | Phagemid | pLit-cI_opt_ | Brodel et al. [2] | 80852 |
| pLITMUS-rpoN-cI4A5C6G7G-J23106-geneIII | Phagemid | pLit-cI_4A5C6G7G_ | Brodel et al. [2] | 80909 |
| pLITMUS-rpoN-cI4A5T6T-J23106-geneIII | Phagemid | pLit-cI_4A5T6T_ | Brodel et al. [2] | 80908 |
| pLITMUS-rpoN-cI5G6T-J23106-geneIII | Phagemid | pLit-cI_5G6T_ | Brodel et al. [2] | 80907 |
| pLITMUS-rpoN-cI5G6G-J23106-geneIII | Phagemid | pLit-cI_5G6G_ | Brodel et al. [2] | 80906 |
| pLITMUS-rpoN-cI5C6A-J23106-geneIII | Phagemid | pLit-cI_5C6A_ | Brodel et al. [2] | 80905 |
| pLITMUS-rpoN-cI-J23106-geneIII | Phagemid | pLit-cI_WT_ | Brodel et al. [2] | 80843 |
| pLITMUS*-PrpoN-CroAct3-PBBa_J23106-geneIII | Phagemid | pLit*-cro | Brodel et al. [16] | 134354 |
| M13KO7-ΔPS-ΔgeneIII | Helper | Helper plasmid | Brodel et al. [16] | 134351 |
| pJPC12-ΔM13-mCherry-P/PM,4A5T6T-GFP | Reporter | RP_4A5C6G7G_ | Brodel et al. [2] | 80913 |
| pJPC12-ΔM13-mCherry-P/PM,5G6T-GFP | Reporter | RP_4A5T6T_ | Brodel et al. [2] | 80912 |
| pJPC12-ΔM13-mCherry-P/PM,5G6G-GFP | Reporter | RP_5G6T_ | Brodel et al. [2] | 80911 |
| pJPC12-ΔM13-mCherry-P/PM,5C6A-GFP | Reporter | RP_5G6G_ | Brodel et al. [2] | 80910 |
| pJPC12-ΔM13-mCherry-P/PM,4A5C6G7G-GFP | Reporter | RP_5C6A_ | Brodel et al. [2] | 80914 |
| pJPC12-ΔM13-mCherry-PR/PRM-GFP | Reporter | RP_WT_ | Brodel et al. [2] | 80859 |

**Supplementary Table 2: Genotypes of *E. coli* strains used in this study.**

| **Strain** | **Genotype** | **Company** |
| --- | --- | --- |
| TG1 | F’[traD36 lacIq ∆(lacZ) M15 proA+B+] glnV (supE) thi-1 ∆(mcrB-hsdSM)5 (rK- mK- McrB- ) thi ∆(lac proAB) | Zymo Research |
| TOP10 | F- mcrA Δ( mrr-hsdRMS-mcrBC) Φ80lacZΔM15 Δ lacX74 recA1 araD139 Δ(araleu)7697 galU galK rpsL (StrR) endA1 nupG | Thermo Fisher Scientific |

**Supplementary Table 3: Primers used for cloning, sequencing, and qPCR in this study.**

| **Name** | **Oligonucleotide Sequence** | **Use** | **Details** |
| --- | --- | --- | --- |
| ABB2021_7 | CTGGCCTGCAGTAAAGCC | Cloning | Forward primer pLit* backbone |
| ABB2021_8 | GCAAAAGGCCAGCAAAAGG | Cloning | Reverse primer pLit* backbone |
| ABB2021_9 | TCCTGGCCTTTTGCTGGC | Cloning | Forward primer *cI* fragment in Plit backbone |
| ABB2021_10 | CGAAGCGGGCTTTACTGC | Cloning | Reverse primer *cI* fragment in Plit backbone |
| BD2 | TTAATGCGCCGCTACAGG | Sequencing | Sequencing at pLit ori mutation site |
| BD1 | GATCGTGAGATAGGTGCC | Sequencing | Sequencing at pLit *gIII* RBS mutation site |
| birA_FW | ACGACACTCTCTTCAACACG | qPCR | Forward primer qPCR, amplicon 180bp |
| birA_RV | AGTATTACGCAAGCTGGGTG | qPCR | Reverse primer qPCR, amplicon 180bp |
| bla_FW | CTTCTGACAACGATCGGAGG | qPCR | Forward primer qPCR, amplicon 101bp |
| bla_RV | TCATTCAGCTCCGGTTCCC | qPCR | Reverse primer qPCR, amplicon 101bp |
| pET-birA_FW | atctgcacccagcttgcgtaatact CAGAAGGCCATCCTGACG | Cloning | Cloning pET-birA plasmid (containing both bir and bla sites used for standard curves in qPCR) |
| pET-birA_RV | tgcgccgtgttgaagagagtgtcgt CCAATTGACTGGCCGTCG | Cloning | Cloning pET-birA plasmid (containing both bir and bla sites used for standard curves in qPCR) |

******

**Supplementary Figure 1: Sequences of synthetic promoters in reporter plasmids.** Synthetic promoters were derived by Brodel et al. [2], from the consensus sequence of bacteriophage λ operators, and inserted into bidirectional promoters P_R_/P_RM_. The promoters (as well as the corresponding cIλ variants) are named after the positions of base substitutions within the consensus sequences. The -10 and -35 RNAP promoter regions are unaltered in all synthetic promoters (underlined). Operator regions are highlighted: OR1 blue, OR2 red, OR3 green. Mutated nucleotide base pairs in OR1 and OR2 of the synthetic promoters are in bold. Obliterated (purple) bases in OR3 in all synthetic promoters are present to prevent cIλ binding, and to provide RNAP binding access. The natural P_R_/P_RM_ sequence is shown for non-obliterated OR3 comparison (bottom).

**cI**

ATGAGCACAAAAAAGAAACCATTAACACAAGAGCAGCTTGAGGACGCACGTCGCCTTAAAGCAATTTATGAAAAAAAGAAAAATGAACTTGGCTTATCCCAGGAATCTGTCGCAGACAAGATGGGGATGGGGCAGTCAGGCGTTGGTGCTTTATTTAATGGCATCAATGCATTAAATGCTTATAACGCCGCATTGCTTGCAAAAATTCTCAAAGTTAGCGTTGAAGAATTTAGCCCTTCAATCGCCAGAGAAATCTACGAGATGTATGAAGCGGTTAGTATGCAGCCGTCACTTAGAAGTGAGTATGAGTACCCTGTTTTTTCTCATGTTCAGGCAGGGATGTTCTCACCTGAGCTTAGAACCTTTACCAAAGGTGATGCGGAGAGATGGGTAAGCACAACCAAAAAAGCCAGTGATTCTGCATTCTGGCTTGAGGTTGAAGGTAATTCCATGACCGCACCAACAGGCTCCAAGCCAAGCTTTCCTGACGGAATGTTAATTCTCGTTGACCCTGAGCAGGCTGTTGAGCCAGGTGATTTCTGCATAGCCAGACTTGGGGGTGATGAGTTTACCTTCAAGAAACTGATCAGGGATAGCGGTCAGGTGTTTTTACAACCACTAAACCCACAGTACCCAATGATCCCATGCAATGAGAGTTGTTCCGTTGTGGGGAAAGTTATCGCTAGTCAGTGGCCTGAAGAGACGTTTGGCTGA

**cI_opt_**

ATGAGCACAAAAAAGAAACCATTAACACAAGAGCAGCTTGAGGACGCACGTCGCCTTAAAGCAATTTATGAAAAAAAGAAAAATGAACTTGGCTTATCCCAGGAATTGGTCGCATACGAGATGGGGATGGGGCAGTCAGGCGTTGGTGCTTTATTTAATGGCATCAATGCATTAAATGCTTATAACGCCGCATTGCTTGCAAAAATTCTCAAAGTTAGCGTTGAAGAATTTAGCCCTTCAATCGCCAGAGAAATCTACGAGATGTATGAAGCGGTTAGTATGCAGCCGTCACTTAGAAGTGAGTATGAGTACCCTGTTTTTTCTCATGTTCAGGCAGGGATGTTCTCACCTGAGCTTAGAACCTTTACCAAAGGTGATGCGGAGAGATGGGTAAGCACAACCAAAAAAGCCAGTGATTCTGCATTCTGGCTTGAGGTTGAAGGTAATTCCATGACCGCACCAACAGGCTCCAAGCCAAGCTTTCCTGACGGAATGTTAATTCTCGTTGACCCTGAGCAGGCTGTTGAGCCAGGTGATTTCTGCATAGCCAGACTTGGGGGTGATGAGTTTACCTTCAAGAAACTGATCAGGGATAGCGGTCAGGTGTTTTTACAACCACTAAACCCACAGTACCCAATGATCCCATGCAATGAGAGTTGTTCCGTTGTGGGGAAAGTTATCGCTAGTCAGTGGCCTGAAGAGACGTTTGGCTGA

**cI_5C6A_**

ATGAGCACAAAAAAGAAACCATTAACACAAGAGCAGCTTGAGGACGCACGTCGCCTTAAAGCAATTTATGAAAAAAAGAAAAATGAACTTGGCTTATCCCAGGAATCTGTCGCAGACAAGATGGGGATGGGGCAGGGGAGGGTGAGTGCTTTATTTAATGGCATCGTGGCATTAAATGCTTATAACGCCGCATTGCTTGCAAAAATTCTCAAAGTTAGCGTTGAAGAATTTAGCCCTTCAATCGCCAGAGAAATCTACGAGATGTATGAAGCGGTTAGTATGCAGCCGTCACTTAGAAGTGAGTATGAGTACCCTGTTTTTTCTCATGTTCAGGCAGGGATGTTCTCACCTGAGCTTAGAACCTTTACCAAAGGTGATGCGGAGAGATGGGTAAGCACAACCAAAAAAGCCAGTGATTCTGCATTCTGGCTTGAGGTTGAAGGTAATTCCATGACCGCACCAACAGGCTCCAAGCCAAGCTTTCCTGACGGAATGTTAATTCTCGTTGACCCTGAGCAGGCTGTTGAGCCAGGTGATTTCTGCATAGCCAGACTTGGGGGTGATGAGTTTACCTTCAAGAAACTGATCAGGGATAGCGGTCAGGTGTTTTTACAACCACTAAACCCACAGTACCCAATGATCCCATGCAATGAGAGTTGTTCCGTTGTGGGGAAAGTTATCGCTAGTCAGTGGCCTGAAGAGACGTTTGGCTGA

**Supplementary Figure 2:** Gene sequences of *cIλ* variants encoded in the phagemids remastered in this study. Mutations from wild-type *cI* for DNA-binding specificity are highlighted green, and mutations to wild-type *cI* for stronger activation activity (RNA polymerase recruitment; Bushman *et al.* (1989)) are highlighted blue.

**cI_5C6A,P_**

ATGAGCACAAAAAAGAAACCATTAACACAAGAGCAGCTTGAGGACGCACGTCGCCTTAAAGCAATTTATGAAAAAAAGAAAAATGAACTTGGCTTATCCCAGGAATTGGTCGCATACGAGATGGGGATGGGGCAGGGGAGGGTGAGTGCTTTATTTAATGGCATCGTGGCATTAAATGCTTATAACGCCGCATTGCTTGCAAAAATTCTCAAAGTTAGCGTTGAAGAATTTAGCCCTTCAATCGCCAGAGAAATCTACGAGATGTATGAAGCGGTTAGTATGCAGCCGTCACTTAGAAGTGAGTATGAGTACCCTGTTTTTTCTCATGTTCAGGCAGGGATGTTCTCACCTGAGCTTAGAACCTTTACCAAAGGTGATGCGGAGAGATGGGTAAGCACAACCAAAAAAGCCAGTGATTCTGCATTCTGGCTTGAGGTTGAAGGTAATTCCATGACCGCACCAACAGGCTCCAAGCCAAGCTTTCCTGACGGAATGTTAATTCTCGTTGACCCTGAGCAGGCTGTTGAGCCAGGTGATTTCTGCATAGCCAGACTTGGGGGTGATGAGTTTACCTTCAAGAAACTGATCAGGGATAGCGGTCAGGTGTTTTTACAACCACTAAACCCACAGTACCCAATGATCCCATGCAATGAGAGTTGTTCCGTTGTGGGGAAAGTTATCGCTAGTCAGTGGCCTGAAGAGACGTTTGGCTGA

**cI_5G6G_**

ATGAGCACAAAAAAGAAACCATTAACACAAGAGCAGCTTGAGGACGCACGTCGCCTTAAAGCAATTTATGAAAAAAAGAAAAATGAACTTGGCTTATCCCAGGAATCTGTCGCAGACAAGATGGGGATGGGGCAGTCCGCGGTTTCCGAGTTATTTAATGGCATCTGGGCATTAAATGCTTATAACGCCGCATTGCTTGCAAAAATTCTCAAAGTTAGCGTTGAAGAATTTAGCCCTTCAATCGCCAGAGAAATCTACGAGATGTATGAAGCGGTTAGTATGCAGCCGTCACTTAGAAGTGAGTATGAGTACCCTGTTTTTTCTCATGTTCAGGCAGGGATGTTCTCACCTGAGCTTAGAACCTTTACCAAAGGTGATGCGGAGAGATGGGTAAGCACAACCAAAAAAGCCAGTGATTCTGCATTCTGGCTTGAGGTTGAAGGTAATTCCATGACCGCACCAACAGGCTCCAAGCCAAGCTTTCCTGACGGAATGTTAATTCTCGTTGACCCTGAGCAGGCTGTTGAGCCAGGTGATTTCTGCATAGCCAGACTTGGGGGTGATGAGTTTACCTTCAAGAAACTGATCAGGGATAGCGGTCAGGTGTTTTTACAACCACTAAACCCACAGTACCCAATGATCCCATGCAATGAGAGTTGTTCCGTTGTGGGGAAAGTTATCGCTAGTCAGTGGCCTGAAGAAACGTTTGGCTGA

**cI_5G6G,P_**

ATGAGCACAAAAAAGAAACCATTAACACAAGAGCAGCTTGAGGACGCACGTCGCCTTAAAGCAATTTATGAAAAAAAGAAAAATGAACTTGGCTTATCCCAGGAATTGGTCGCATACGAGATGGGGATGGGGCAGTCCGCGGTTTCCGAGTTATTTAATGGCATCTGGGCATTAAATGCTTATAACGCCGCATTGCTTGCAAAAATTCTCAAAGTTAGCGTTGAAGAATTTAGCCCTTCAATCGCCAGAGAAATCTACGAGATGTATGAAGCGGTTAGTATGCAGCCGTCACTTAGAAGTGAGTATGAGTACCCTGTTTTTTCTCATGTTCAGGCAGGGATGTTCTCACCTGAGCTTAGAACCTTTACCAAAGGTGATGCGGAGAGATGGGTAAGCACAACCAAAAAAGCCAGTGATTCTGCATTCTGGCTTGAGGTTGAAGGTAATTCCATGACCGCACCAACAGGCTCCAAGCCAAGCTTTCCTGACGGAATGTTAATTCTCGTTGACCCTGAGCAGGCTGTTGAGCCAGGTGATTTCTGCATAGCCAGACTTGGGGGTGATGAGTTTACCTTCAAGAAACTGATCAGGGATAGCGGTCAGGTGTTTTTACAACCACTAAACCCACAGTACCCAATGATCCCATGCAATGAGAGTTGTTCCGTTGTGGGGAAAGTTATCGCTAGTCAGTGGCCTGAAGAAACGTTTGGCTGA

**Supplementary Figure 2 (continued):** Gene sequences of *cIλ* variants encoded in the phagemids remastered in this study. Mutations from wild-type *cI* for DNA-binding specificity are highlighted green, and mutations to wild-type *cI* for stronger activation activity (RNA polymerase recruitment; Bushman *et al.* (1989)) are highlighted blue.

**cI_5G6T_**

ATGAGCACAAAAAAGAAACCATTAACACAAGAGCAGCTTGAGGACGCACGTCGCCTTAAAGCAATTTATGAAAAAAAGAAAAATGAACTTGGCTTATCCCAGGAATCTGTCGCAGACAAGATGGGGATGTGGCAGTCCAACTTGTCGGCTTTATTTAATGGCATCTCAGCATTAAATGCTTATAACGCCGCATTGCTTGCAAAAATTCTCAAAGTTAGCGTTGAAGAATTTAGCCCTTCAATCGCCAGAGAAATCTACGAGATGTATGAAGCGGTTAGTATGCAGCCGTCACTTAGAAGTGAGTATGAGTACCCTGTTTTTTCTCATGTTCAGGCAGGGATGTTCTCACCTGAGCTTAGAACCTTTACCAAAGGTGATGCGGAGAGATGGGTAAGCACAACCAAAAAAGCCAGTGATTCTGCATTCTGGCTTGAGGTTGAAGGTAATTCCATGACCGCACCAACAGGCTCCAAGCCAAGCTTTCCTGACGGAATGTTAATTCTCGTTGACCCTGAGCAGGCTGTTGAGCCAGGTGATTTCTGCATAGCCAGACTTGGGGGTGATGAGTTTACCTTCAAGAAACTGATCAGGGATAGCGGTCAGGTGTTTTTACAACCACTAAACCCACAGTACCCAATGATCCCATGCAATGAGAGTTGTTCCGTTGTGGGGAAAGTTATCGCTAGTCAGTGGCCTGAAGAGACGTTTGGCTGA

**cI_5G6T,P_**

ATGAGCACAAAAAAGAAACCATTAACACAAGAGCAGCTTGAGGACGCACGTCGCCTTAAAGCAATTTATGAAAAAAAGAAAAATGAACTTGGCTTATCCCAGGAATTGGTCGCATACGAGATGGGGATGTGGCAGTCCAACTTGTCGGCTTTATTTAATGGCATCTCAGCATTAAATGCTTATAACGCCGCATTGCTTGCAAAAATTCTCAAAGTTAGCGTTGAAGAATTTAGCCCTTCAATCGCCAGAGAAATCTACGAGATGTATGAAGCGGTTAGTATGCAGCCGTCACTTAGAAGTGAGTATGAGTACCCTGTTTTTTCTCATGTTCAGGCAGGGATGTTCTCACCTGAGCTTAGAACCTTTACCAAAGGTGATGCGGAGAGATGGGTAAGCACAACCAAAAAAGCCAGTGATTCTGCATTCTGGCTTGAGGTTGAAGGTAATTCCATGACCGCACCAACAGGCTCCAAGCCAAGCTTTCCTGACGGAATGTTAATTCTCGTTGACCCTGAGCAGGCTGTTGAGCCAGGTGATTTCTGCATAGCCAGACTTGGGGGTGATGAGTTTACCTTCAAGAAACTGATCAGGGATAGCGGTCAGGTGTTTTTACAACCACTAAACCCACAGTACCCAATGATCCCATGCAATGAGAGTTGTTCCGTTGTGGGGAAAGTTATCGCTAGTCAGTGGCCTGAAGAGACGTTTGGCTGA

**cI_4A5T6T_**

ATGAGCACAAAAAAGAAACCATTAACACAAGAGCAGCTTGAGGACGCACGTCGCCTTAAAGCAATTTATGAAAAAAAGAAAAATGAACTTGGCTTATCCCAGGAATCTGTCGCAGACAAGATGGGGATGTGGCAGAACCGCATCTGCGCTTTATTTAATGGCATCGCGGCATTAAATGCTTATAACGCCGCATTGCTTGCAAAAATTCTCAAAGTTAGCGTTGAAGAATTTAGCCCTTCAATCGCCAGAGAAATCTACGAGATGTATGAAGCGGTTAGTATGCAGCCGTCACTTAGAAGTGAGTATGAGTACCCTGTTTTTTCTCATGTTCAGGCAGGGATGTTCTCACCTGAGCTTAGAACCTTTACCAAAGGTGATGCGGAGAGATGGGTAAGCACAACCAAAAAAGCCAGTGATTCTGCATTCTGGCTTGAGGTTGAAGGTAATTCCATGACCGCACCAACAGGCTCCAAGCCAAGCTTTCCTGACGGAATGTTAATTCTCGTTGACCCTGAGCAGGCTGTTGAGCCAGGTGATTTCTGCATAGCCAGACTTGGGGGTGATGAGTTTACCTTCAAGAAACTGATCAGGGATAGCGGTCAGGTGTTTTTACAACCACTAAACCCACAGTACCCAATGATCCCATGCAATGAGAGTTGTTCCGTTGTGGGGAAAGTTATCGCTAGTCAGTGGCCTGAAGAGACGTTTGGCTGA

**Supplementary Figure 2 (continued):** Gene sequences of *cIλ* variants encoded in the phagemids remastered in this study. Mutations from wild-type *cI* for DNA-binding specificity are highlighted green, and mutations to wild-type *cI* for stronger activation activity (RNA polymerase recruitment; Bushman *et al.* (1989)) are highlighted blue.

**cI_4A5T6T,P_**

ATGAGCACAAAAAAGAAACCATTAACACAAGAGCAGCTTGAGGACGCACGTCGCCTTAAAGCAATTTATGAAAAAAAGAAAAATGAACTTGGCTTATCCCAGGAATTGGTCGCATACGAGATGGGGATGTGGCAGAACCGCATCTGCGCTTTATTTAATGGCATCGCGGCATTAAATGCTTATAACGCCGCATTGCTTGCAAAAATTCTCAAAGTTAGCGTTGAAGAATTTAGCCCTTCAATCGCCAGAGAAATCTACGAGATGTATGAAGCGGTTAGTATGCAGCCGTCACTTAGAAGTGAGTATGAGTACCCTGTTTTTTCTCATGTTCAGGCAGGGATGTTCTCACCTGAGCTTAGAACCTTTACCAAAGGTGATGCGGAGAGATGGGTAAGCACAACCAAAAAAGCCAGTGATTCTGCATTCTGGCTTGAGGTTGAAGGTAATTCCATGACCGCACCAACAGGCTCCAAGCCAAGCTTTCCTGACGGAATGTTAATTCTCGTTGACCCTGAGCAGGCTGTTGAGCCAGGTGATTTCTGCATAGCCAGACTTGGGGGTGATGAGTTTACCTTCAAGAAACTGATCAGGGATAGCGGTCAGGTGTTTTTACAACCACTAAACCCACAGTACCCAATGATCCCATGCAATGAGAGTTGTTCCGTTGTGGGGAAAGTTATCGCTAGTCAGTGGCCTGAAGAGACGTTTGGCTGA

**cI_4A5C6G7G_**

ATGAGCACAAAAAAGAAACCATTAACACAAGAGCAGCTTGAGGACGCACGTCGCCTTAAAGCAATTTATGAAAAAAAGAAAAATGAACTTGGCTTATCCCAGGAATCTGTCGCAGACAAGATGGGGATGGGGCAGGCGCGCGTTTCGGAGTTATTTAATGGCATCATGGCATTAAATGCTTATAACGCCGCATTGCTTGCAAAAATTCTCAAAGTTAGCGTTGAAGAATTTAGCCCTTCAATCGCCAGAGAAATCTACGAGATGTATGAAGCGGTTAGTATGCAGCCGTCACTTAGAAGTGAGTATGAGTACCCTGTTTTTTCTCATGTTCAGGCAGGGATGTTCTCACCTGAGCTTAGAACCTTTACCAAAGGTGATGCGGAGAGATGGGTAAGCACAACCAAAAAAGCCAGTGATTCTGCATTCTGGCTTGAGGTTGAAGGTAATTCCATGACCGCACCAACAGGCTCCAAGCCAAGCTTTCCTGACGGAATGTTAATTCTCGTTGACCCTGAGCAGGCTGTTGAGCCAGGTGATTTCTGCATAGCCAGACTTGGGGGTGATGAGTTTACCTTCAAGAAACTGATCAGGGATAGCGGTCAGGTGTTTTTACAACCACTAAACCCACAGTACCCAATGATCCCATGCAATGAGAGTTGTTCCGTTGTGGGGAAAGTTATCGCTAGTCAGTGGCCTGAAGAGACGTTTGGCTGA

**cI_4A5C6G7G,P_**

ATGAGCACAAAAAAGAAACCATTAACACAAGAGCAGCTTGAGGACGCACGTCGCCTTAAAGCAATTTATGAAAAAAAGAAAAATGAACTTGGCTTATCCCAGGAATTGGTCGCATACGAGATGGGGATGGGGCAGGCGCGCGTTTCGGAGTTATTTAATGGCATCATGGCATTAAATGCTTATAACGCCGCATTGCTTGCAAAAATTCTCAAAGTTAGCGTTGAAGAATTTAGCCCTTCAATCGCCAGAGAAATCTACGAGATGTATGAAGCGGTTAGTATGCAGCCGTCACTTAGAAGTGAGTATGAGTACCCTGTTTTTTCTCATGTTCAGGCAGGGATGTTCTCACCTGAGCTTAGAACCTTTACCAAAGGTGATGCGGAGAGATGGGTAAGCACAACCAAAAAAGCCAGTGATTCTGCATTCTGGCTTGAGGTTGAAGGTAATTCCATGACCGCACCAACAGGCTCCAAGCCAAGCTTTCCTGACGGAATGTTAATTCTCGTTGACCCTGAGCAGGCTGTTGAGCCAGGTGATTTCTGCATAGCCAGACTTGGGGGTGATGAGTTTACCTTCAAGAAACTGATCAGGGATAGCGGTCAGGTGTTTTTACAACCACTAAACCCACAGTACCCAATGATCCCATGCAATGAGAGTTGTTCCGTTGTGGGGAAAGTTATCGCTAGTCAGTGGCCTGAAGAGACGTTTGGCTGA

**Supplementary Figure 2 (continued):** Gene sequences of *cIλ* variants encoded in the phagemids remastered in this study. Mutations from wild-type *cI* for DNA-binding specificity are highlighted green, and mutations to wild-type *cI* for stronger activation activity (RNA polymerase recruitment; Bushman *et al.* (1989)) are highlighted blue.

**Supplementary Table 4: Phage titres of pLit*/HP-containing cells in phage production assays, calculated in colony forming units per ml (cfu/ml).**

| **Plasmids Transfected** | | **cfu/ml** |
| --- | --- | --- |
| **Helper** | **Phagemid** |  |
| Y | pLit*-cI5C6A,P | 4.88×10^12^ |
| Y | pLit*-cI5G6T,P | 7.50×10^12^ |
| Y | pLit*-cI4A5T6T,P | 3.72×10^12^ |
| Y | pLit*-cI5G6G,P | 4.46×10^12^ |
| Y | pLit*-cI4A5C6G7G,P | 3.72×10^12^ |
| Y | pLit*-cIopt | 3.54×10^12^ |
| Y | pLit-cI5C6A,P | 4.32×10^12^ |
| Y | pLit-cI4A5C6G7G,P | 4.00×10^12^ |
| Y | pLit*-cI5C6A | 4.90×10^11^ |
| Y | pLit*-cI5G6T | 2.7×10^11^ |
| Y | pLit*-cI4A5T6T | 3.8×10^8^ |
| Y | pLit*-cI5G6G | 2.5×10^11^ |
| Y | pLit*-cI4A5C6G7G | 3.00×10^8^ |
| Y | pLit*-cIWT | 3.30×10^9^ |
| Y | pLit-cI5C6A | 3.40×10^12^ |
| Y | pLit-cI4A5C6G7G | 5.40×10^11^ |
| N | pLit*-cI5C6A | 0.00 |
| Y | N | 0.00 |


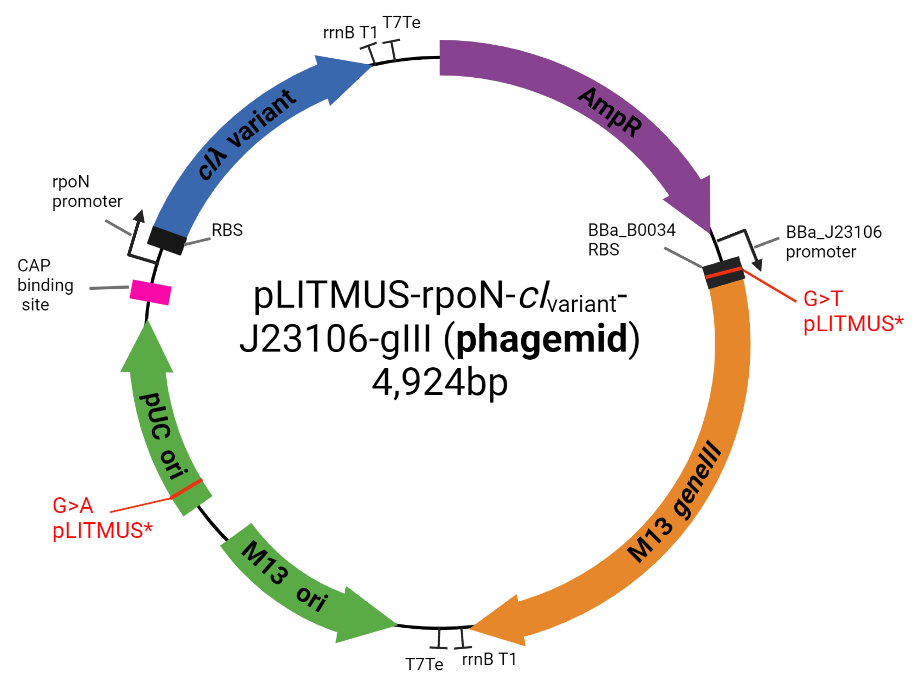

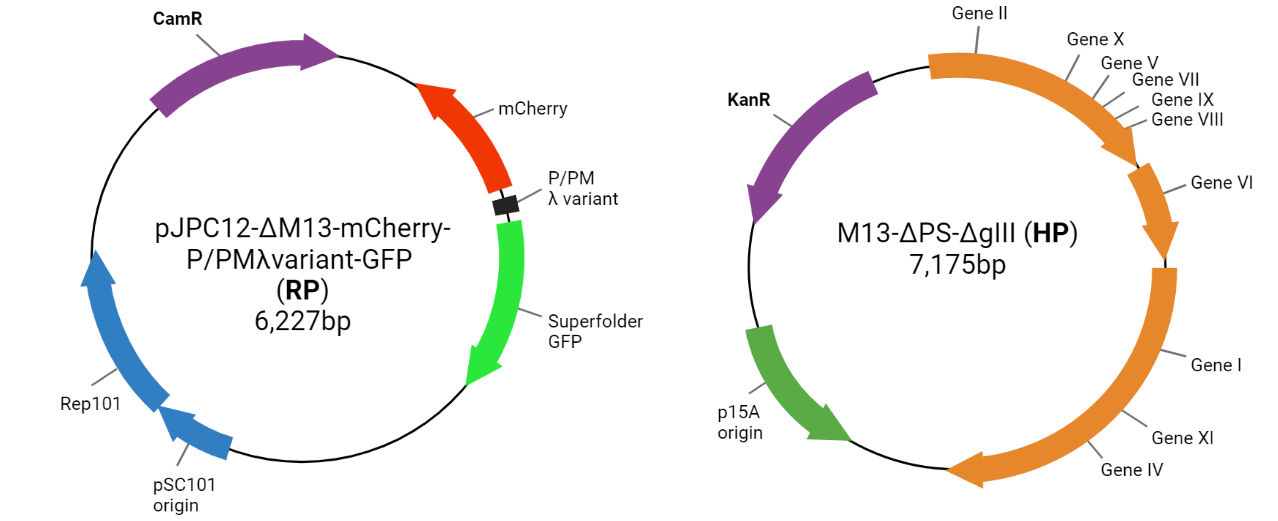


**Supplementary Figure 3: Plasmid maps of the three classes used in this study; phagemid (PM), helper plasmid (HP), and reporter plasmid (RP).**


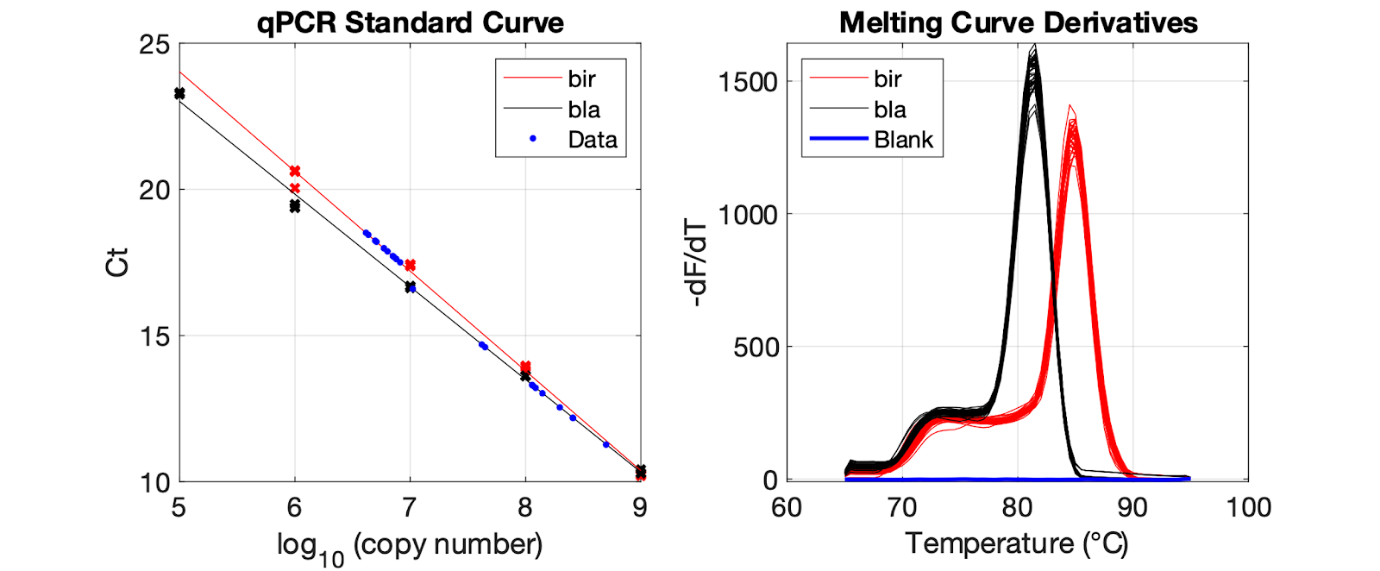


**Supplementary Figure 4: Standard and melting curves for bir and bla primer pairs, obtained for phagemid copy number qPCR analysis.** Standard curves were obtained with 10-fold serial dilutions of the pBR322 plasmid containing bir and bla sites ranging from 10^9^ and 10^5^ copies in the qPCR reaction. Given its size of 3120 bp, 10^9^ copies are present in 3.42 ng DNA. Each standard dilution was amplified with both primer sets in triplicate. The Ct values were plotted against the logarithm of their initial copy numbers. Linear regression models were fit to the standard curves (R > 0.997). The melting curve derivatives were examined to confirm the specificity of the qPCR reactions. The peak melting temperatures were 84.5°C for the bir set and 81.5°C for the bla set. These are consistent with the amplicon sizes, where the bir set has a longer 180 bp product, whereas the bla set has a 101 bp product. The identity of the amplicons was also verified with gel electrophoresis and by sequencing (data not shown). The blue curve corresponds to a blank sample where DNA was replaced with water, where no product is detected, showing lack of contamination.


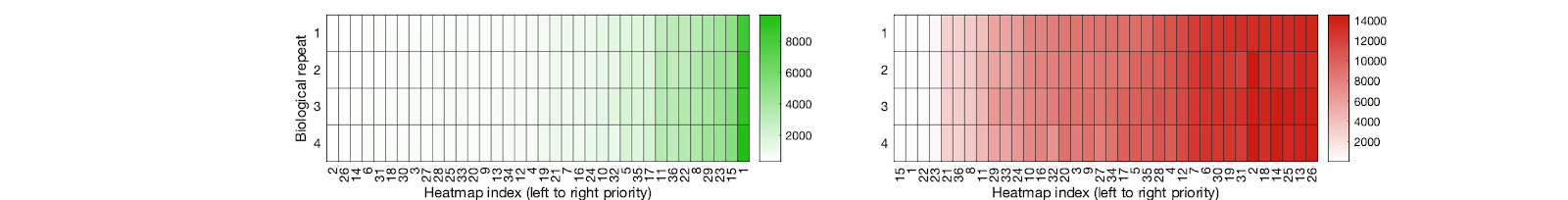

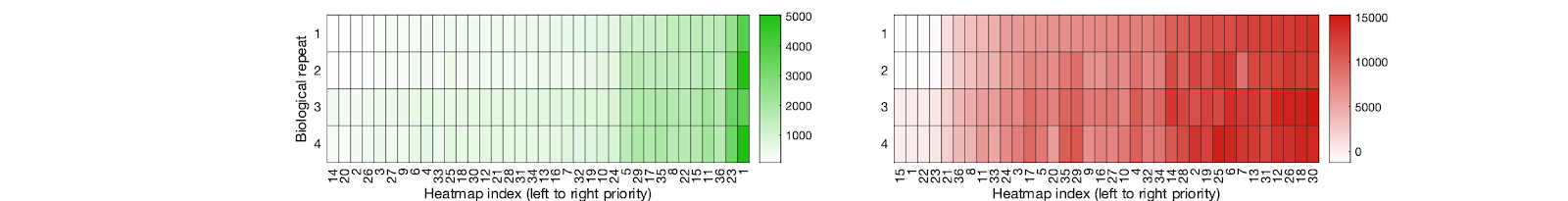


**Supplementary Figure 5: GFP and RFP fluorescence heatmaps from reporter assays showing variability between biological repeats.** The top panels show end point (20hrs) GFP and RFP fluorescence data across the four biological repeats from the six optimised *cI* variants, and the bottom panels show the same but for the six wildtype backbone *cI* variants. The background-subtracted, absorbance-normalised readings of GFP and RFP are plotted (arbitrary units). The 36 boxes correspond to each phagemid/RP combination assayed, as in Figure 5C. The data is arranged in increasing order within the first biological repeat. There is a good correspondence of the measurements between the repeats.
